# Advances on the Antioxidant Peptides from Nuts: A Narrow Review

**DOI:** 10.3390/antiox11102020

**Published:** 2022-10-12

**Authors:** Fanrui Zhao, Chunlei Liu, Laura Bordoni, Irene Petracci, Dan Wu, Li Fang, Ji Wang, Xiyan Wang, Rosita Gabbianelli, Weihong Min

**Affiliations:** 1 School of Food Science and Engineering, Jilin Agricultural University, Changchun 130118, China; 2 School of Advanced Studies, University of Camerino, 62032 Camerino, Italy; 3 National Engineering Laboratory of Wheat and Corn Deep Processing, Changchun 130118, China; 4 Unit of Molecular Biology and Nutrigenomics, School of Pharmacy, University of Camerino, 62032 Camerino, Italy

**Keywords:** nut-derived antioxidant peptide, bioavailability, preparation process, evaluation method, structure-activity relationship

## Abstract

Antioxidant peptides extracted from natural foods have been studied for their potential use in the development of additives, nutraceuticals, and therapeutic agents. Nut proteins are considered an excellent source of plant-derived proteins for the human diet, due to their high protein content and digestibility of up to 86.22%. Furthermore, compared with grain and soybean proteins, nut proteins have a special amino acid composition, which makes their protein structure different, and promotes their disparate functional characteristics and great bioactivity potential. This review presents the most remarkable studies on antioxidant peptides from nuts, to gain insights into feasible production methods, different evaluation indexes within in vivo or in vitro systems, high bioavailability, and the complex structure-activity relationship resulting from the particularity of their protein structure and amino acid composition. Previously published studies mainly focused on the effects of the production methods/processes of nut-derived peptides on antioxidant activity, and proved that nut-extracted antioxidant peptides can resist the degradation of acid, alkali, and gastrointestinal enzymes, have high antioxidant activity in vitro and in vivo, and also have the potential to cross small intestinal epithelial cells in a stable and integral manner. However, the structure-activity relationship of antioxidant peptides from nuts has not been fully established, and the structure information of antioxidant peptides obtained from various nut protein sources is still unclear. The findings presented in this review can be used to provide the theoretical basis for the design and production of nut-derived antioxidant peptides.

## 1. Introduction

**Figure 1:**
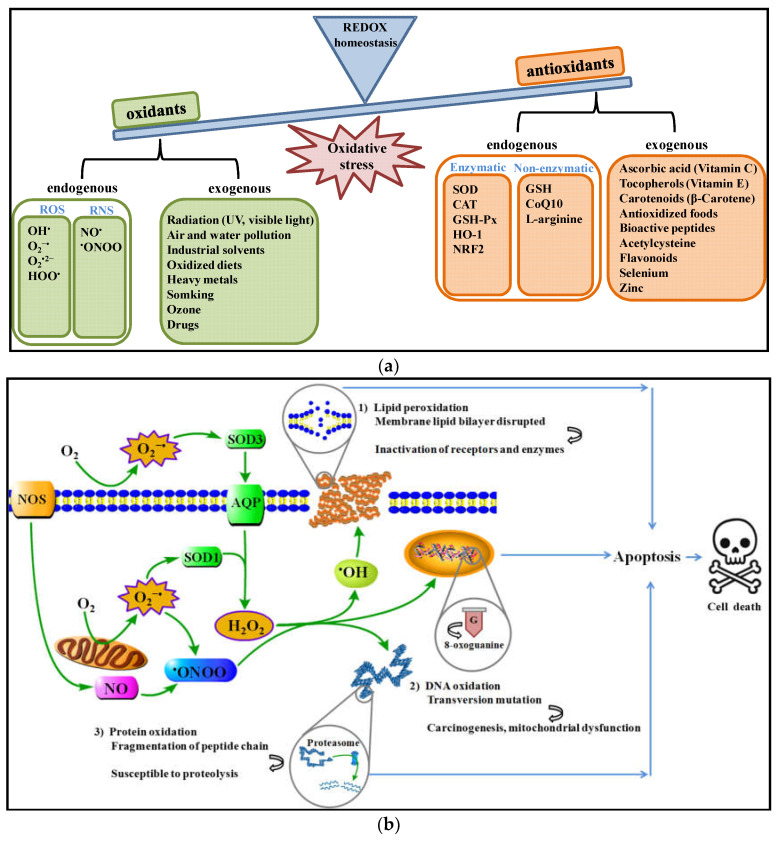
Basic concepts and components of oxidative stress. (**a**) Oxidative stress depends on the imbalance between free radicals and antioxidant systems; (**b**) the effect of oxidative damage on bio-molecules of DNA, proteins, and lipids. Abbreviation: REDOX, oxidation-reduction; ROS, reactive oxygen species; RNS, reactive nitrogen species; •OH, hydroxyl radical; O2−•, superoxide anion radical; H_2_O_2_, hydrogen peroxide; HOO•, peroxyl; NO•, nitric oxide; •ONOO, peroxynitrite; DNA, deoxyribonucleic acid; SOD, superoxide dismutase; CAT, catalase; GSH-Px, glutathione peroxidase; HO-1, heme oxygenase 1; NRF2, nuclear factor erythroid 2-related factor 2; GSH, glutathione; CoQ10, coenzymeQ 10; AQP, aquaporin; NOS, nitric oxide synthase.

Oxidative stress is caused by an imbalance between the production and consumption of oxidative species, and usually involves reactions between free radicals and molecules of high biological importance [1]. Reactive oxygen species (ROS), including hydroxyl radical (•OH), superoxide anion radical (O_2_−•), hydrogen peroxide (H_2_O_2_) and peroxyl radical (HOO•), and reactive nitrogen species (RNS), including nitric oxide (NO•) and peroxynitrite (•ONOO), are well-known as free radicals produced by endogenous oxidation-reduction (REDOX) reactions in eukaryotes (Figure 1a) [2]. In a biological context, ROS/RNS can promote a series of biological processes [3]. However, in cases of excessive production of ROS/RNS or insufficient endogenous enzymatic and non-enzymatic antioxidant defenses, an extensive oxidation of DNA, protein, and lipid occurs, influencing cell structure and function [4]. Finally, cell death or apoptosis occur, together with tissue damage and disease development (Figure 1b) [5].

In a biological context, antioxidant peptides can be divided into two types: endogenous or exogenous peptides [6]. Examples of endogenous antioxidant peptides include glutathione, carnosine, cyclo (His-Pro), and human tripeptide (Gly-His-Lys). In contrast, exogenous antioxidant peptides come from external or environmental sources, such as synthetic antioxidants and dietary food [6]. Examples of synthetic antioxidant peptides include butylated hydroxy-anisole (BHA) and butylated hydroxyl-toluene (BHT), which show stronger antioxidant activities, but at the same time can induce DNA damage and toxicity [7]. Antioxidant peptides derived from natural sources have attracted increasing interest, due to their potential health benefits of low molecular weight (MW), multiple biology activities, high stability, simple structure, easy absorption, and little or no side effects [8]. Therefore, in recent years, there has been great interest in finding new and safe antioxidant peptides from natural sources to be used in food and medicinal materials, to replace synthetic antioxidants.

Plant proteins play a pivotal role in human nutrition, especially in developing countries where the average protein intake is lower than required [9]. Although nut proteins are a common cause of IgE-mediated allergic reactions worldwide, the crude protein content of raw nuts is as high as 31.1%, and most nut proteins contain 18 kinds of amino acids (except Asn and Gln), of which eight kinds of essential amino acids meet human needs and are high-quality plant proteins [10]. In addition, the pattern of essential amino acids in nut protein is close to the standard, which is 0.48 g/kg per day for adults, recommended by the World Health Organization (WHO), Food and Agriculture Organization (FAO), and United Nations University (UNU) [11,12]. Compared with other plant proteins, nut protein contains higher contents of Arg, Glu and Asp, making it a plant protein with special amino acid composition. In particular, compared with legumes, nut proteins are lower in anti-nutrients, such as tannins and saponins, which inhibit the absorption of certain essential nutrients. Therefore, nut proteins are considered to be a critical source of plant proteins in human food and a plant protein with great development potential, and therefore nut proteins must provide the potential for the utilization of antioxidant peptides.

In this narrative review we focus on: (1) the composition, physicochemical properties and structure of nut proteins; (2) the preparation, purification, and identification methods used to study these peptides; (3) the test of antioxidant activity in vitro and in vivo; (4) the bioaccessibility and bioavailability of antioxidant peptides from nuts; (5) the structure-activity relationship (SAR) of antioxidant peptides.

The search strategy included the terms “oxidative stress AND plant-based peptides” or “endogenous oxidation-reduction” or “composition AND nut proteins” or “digestibility AND nut proteins” or “structure AND nut proteins” or “physicochemical properties AND nut proteins” or “preparation AND nut-derived antioxidant peptides” or “identification AND nut-derived antioxidant peptides” or “in vitro AND nut-derived antioxidant peptides” or “in vivo AND nut-derived antioxidant peptides” or “structure-activity relationship AND nut-derived antioxidant peptides” or “gastrointestinal AND nut-derived antioxidant peptides” as keywords. The search was performed in the NCBI-PubMed database. The records were screened for relevant information, and only papers related to antioxidant peptides of nuts published within the period 2010–2022 were selected and studied. In addition, this review also cited three articles published before 2010 that were closely related to antioxidant detection methods and nut protein composition.

## 2. Composition, Structure, and Function Properties of Nut Protein

Nut proteins are an interesting source, due to their excellent nutritional value and low cost. The nutritional value of nuts is mainly related to protein composition, high protein content, and digestibility, which gives nut proteins a higher nutritional quality than other plant proteins. Nut proteins include albumins (soluble in water and dilute solution), globulins (divided into euglobulin-soluble in dilute solutions, acids and alkalis insoluble in water and pseudoglobulins, moderately soluble in these solutions), prolamins (soluble in a solution containing 50–90% ethanol), glutenins (soluble in dilute acid and alkalis) and scleroproteins (insoluble in all mentioned solvents) [13]. The protein content and composition of nuts are different, according to the nut species, varieties, and processed conditions. As shown in Table 1, the walnut protein content in oil-extraction residue are approximately 55.96%, including glutelins (70.1%), globulins (17.6%), albumins (6.8%) and prolamins (5.3%) [14,15]. After degreasing, the protein content of the cashew flour increases from 20.2% to 40.74% [16], including globulins 17.3%, albumins 7.69%, and glutelins 7.8% [10]. However, the main part of the almond and hazelnut protein is composed of albumins, followed by globulins [13]. The protein percentages of albumin and globulin were 75.43% and 13.63% in almond, 67.18% and 17.62% in hazelnut, respectively [13].

Protein quality is defined based on the amino acid pattern and percent of digestibility of proteins. In vitro protein digestibility was carried out by a version of the method of Kang et al. [17]. The digestibility for nut proteins has been estimated to be approximately above 70% in Table 1, which is higher than that of other plant proteins, such as wheat digestibility which is 46%. Meanwhile, the limiting amino acid of nut total protein is usually Met and Cys. Met plus Cys, Lys and Thr were reported to be the first, second and third limiting essential amino acids in almonds compared with the amino acid pattern recommended by WHO/FAO for children (2 to 5 years old). However, compared with the recommended adult amino acid pattern, only sulfur-containing amino acids (Met and Cys) were limited. Among them, the limiting amino acids of different protein factions are distinct. Among walnut proteins, prolamins have a unique amino acid composition, in that they are rich in branched-chain amino acids and neutral amino acids, which is a rare characteristic found in plant protein. In addition, Lys was the first limiting essential amino acid in globulin and glutenin components, whereas Leu and Met plus Cys were the second limiting essential amino acids in the prolamin and albumin components of walnut, respectively. Lys, Ile and Try were limited in the albumin components of almonds, whereas in the globulin components, Lys and Try were limited [18]. Additionally, Met, Lys and Thr were the first, second, and third limiting amino acids in the globulins of almonds.

In addition to nutritional quality, the functional properties of nut proteins are also an important aspect, such as water solubility, emulsifying ability, and foaming ability. The compositions of amino acids influence the functional properties of nut proteins. Additionally, the structural changes of nut proteins lead to differences in their functional properties, highlighting the close relationship between the amino acid composition, structure and function. Compared with albumin and globulin, glutenin components in cashews have a higher aromatic and hydrophobic amino acid content, which enhances protein–protein interactions and leads to higher oil-holding capacity. Albumin has a low content of aromatic and hydrophobic amino acids, which may explain its high solubility [10]. Other reasons explaining why the glutelin component of cashews has a higher oil-holding capacity and water-holding capacity may be the natural amphiphilic nature and the more open structure, compared with other proteins whose structure contains more β-sheet and turns, which may limit their interactions with water and lipid phases [10]. 

Since most natural proteins do not possess the functional properties required by the food industry, they need to be modified to improve these properties, in particular solubility. The major fraction of proteins in walnuts is glutelins, whose low water solubility limits their functional performance in many aqueous-based food products. However, water solubility (93.2%), emulsifying activity (44.7 m^2^/g), and emulsifying stability (32.2 ± 0.8 min) of walnut protein with a high level of sonication for 15 min were significantly increased compared with that of the untreated control sample (76.4%, 35.4 m^2^/g and 23.0 ± 1.3 min, respectively) [19]. The observed phenomenon was probably associated with the fact that the ultrasonic waves disrupted some of the physical forces holding the protein together in the larger aggregates, thereby releasing smaller soluble proteins. In addition, a secondary structure analysis performed using circular dichroism (CD) indicated that sonication may have caused preferential disruption of certain types of hydrogen bonds, thereby causing some of the α-helix structures to be converted into β-sheet, β-turn, or random coil structures [20,21]. Sonication may also have promoted the breakage of some of the S-S bonds in the proteins, leading to the formation of new S-H bonds, which altered their surface chemistry, and therefore their functional properties.

The various effects observed in studies are probably due to differences in the protein type, processing technology, and condition used. Peanut protein hydrolysates were enzymatically produced through Alcalase 2.4 L under different degrees of hydrolysis (DH), which significantly improved the water solubility compare with peanut protein, especially in the pH range of 4 to 6 [22]. However, peanut protein showed better emulsifying and foaming properties than peanut protein hydrolysate, due to DH reducing the peptide chain length, which led to lower efficiency in reducing the interfacial tension [22]. Another reason is that the change in pH affects the surface charge of the protein or peptide, resulting in the emulsifying properties of protein higher than its enzymatic hydrolysate [23]. The hydrolysis of the peanut protein increases the formation of the foaming ability, especially in the range of pH 3 to 6; however, the formation of the foam stability in this pH condition is poor [22]. This may be because although the smaller peptides are able to incorporate more air into the solution than larger peptides and increase the foaming capacity of the solution, they do not have enough strength to produce stable foam [24]. In addition, the hydrolysis of proteins releases peptides that change polarity or hydrophobicity, which can also affect foaming characteristics. In conclusion, the shorter peptides released from proteins after hydrolysis could improve functional properties by converting proteins into peptides with the desired size, charge, and surface properties.

## 3. Production of Nut-Derived Antioxidant Peptides

The bioactive peptides are inactive when they are part of the parent protein, but turn active when released, due to the action of chemical, enzymatic and microbial fermentation methods. Bioactive peptides are more easily absorbed by the body, and exert various bioactive activities [25]. Compared with chemical and microbial fermentation methods, enzymatic process is preferred when producing peptides, as other methods can leave residual organic solvents or toxic chemicals in the final products [9]. The commonly used hydrolases are endogenous enzymes (such as trypsin, pepsin, and pancreatin) and exogenous enzymes, including neutral, flavourzyme, Alcalase, papain, and bromelain. Various nut-derived peptides with biological activities such as antibacterial, anti-inflammatory, and antioxidant activity are obtained by enzymatic hydrolysis. Among them, the antioxidant activity of nut-derived peptides is a major interest of researchers all over the world.

Table 2 summarizes selected examples of enzymatic hydrolysis in studies that successfully identified antioxidant peptides from the nut proteins. Different enzymes have different specificities for the cleavage of individual patterns of the peptide bonds [5]. For example, papain cuts the peptide bonds of hydrophobic regions that include the amino acids Ala, Val, Leu, Ile, Phe, Trp, and Tyr [5]. In the presence of Arg or Lys, trypsin specifically cleaves the C-terminal peptide bonds. Furthermore, pancreatin preferentially cleaves the peptide bonds of the N-terminal phosphorylated regions and the C-terminal hydrophobic regions [26]. Alcalase shows broader specificity than other enzymes, due to the production of peptides containing Glu, Met, Leu, Tyr, Lys, and Gln. Pepsin cleaves the peptide bond after Phe and Leu. In some instances, two or more different proteases were combined to produce the desired nut proteins and nut by-products hydrolysate [27]. Wang et al. reported walnut hydrolysates with high antioxidant activity (ROS levels decreased) by adding complex Viscozyme L and pancreatin at a protease/substrate ratio of 1.0% (*w*/*w*) at pH 7.0, 55 °C for 12 h [28]. Therefore, the type of protease is the main factor in obtaining peptides, and ultimately affects the antioxidant activity of hydrolysates. Moreover, it is also important to have appropriate conditions for the hydrolysate production, such as enzyme/substrate ratio, temperature, pH, and time. Generally, in the process of enzymatic hydrolysis, the DH and the yield of proteins are considered important parameters for evaluating the results of enzymatic hydrolysis. Ren et al. obtained the peptides with the highest antioxidant activity when a walnut protein isolate with 32.23% DH was hydrolyzed using neutrase (9000 U/g) at pH 7.0 and 52.5 °C and using Alcalase (7000 U/g) at pH 8.4 and 55.5 °C for 3 h, separately [26]. The DH is not directly related to the antioxidant capacity of the peptides; it is only indicative of the average molecular size of the peptides present in a hydrolysate. The antioxidant property of peptides is dependent not only on their molecular size, but also on the amino acids that compose them, and their position in the chain. In brief, these methods provided a simple and convenient way for the hydrolysis of nut proteins, leading to the formation of hydrolysates with antioxidative properties.

## 4. Approaches for Measuring Antioxidant Capacity

Antioxidants exert their activity through two main mechanisms: hydrogen transfer and electron donation [5]. Hydrogen donation is more relevant in the context of chain-breaking reactions, which are usually completed in a matter of minutes. Electron transfer, on the other hand, involves a probe that changes its maximum absorbance in response to antioxidants, which is also a relatively simple and fast reaction [5]. Basically, the methods used to evaluate the antioxidant activity after enzymatic hydrolysis could be broadly divided into in silico, in vitro, in vivo, and ex vivo, whereas the identification and assessment of purity are mostly used in vitro (cell models) and in vivo (animal models) assays. However, usually more than two assays are commonly used to determine the antioxidant activity, because there is no strict detection method or standard for the characterization of the overall antioxidant potential of protein hydrolysates, partially purified peptides, and individual peptides. 

### 4.1. Chemical Assays

The chemical assay is the most important method applied to characterize the antioxidant capacity of the enzymatic hydrolysis and purification identification from nut protein hydrolysates. As shown in Table 3, the most widely used chemical analysis used to evaluate the antioxidant properties of protein hydrolysates and peptides derived from nut proteins, are (1) oxygen radical absorbance capacity (ORAC); (2) ferric reducing antioxidant power (FRAP); (3) 2,2-diphenyl-1-picrylhydrazyl (DPPH); (4) 2,2′-azino-bis-(3-ethylbenzothiazoline-6-sulphonic acid) (ABTS); (5) metal chelating activity (MCA); (6) hydroxyl radical scavenging activity; (7) superoxide radical scavenging assay (SRSA). The mechanisms underlying each chemical assay used to evaluate the antioxidant properties are different. For example, DPPH assay is based on the theory that the hydrogen donor is an antioxidant, in which the mechanism is DPPH• which accepts hydrogen from an antioxidant [57]. In ABTS assay, a stable ABTS radical cation, which has a blue-green chromophore absorption, was produced by the oxidation of ABTS with potassium persulfate, prior to the addition of antioxidants [58]. The antioxidant effect (reducing ability) can be evaluated by monitoring the formation of a Fe2+-TPTZ complex with a spectrophotometer in the FRAP assay [59]. Therefore, assays developed to evaluate the antioxidant activity of nut-derived protein hydrolysates and peptides vary. Chen et al. reported that the hydrolysates of defatted walnut meal showed high hydroxyl radical scavenging activity (44.14% at 1.0 mg/mL) and ORAC value (1428.13 μmol Trolox Equivalent/g), equivalent to glutathione (GSH) (the hydroxyl radical scavenging activity was 41.57% and the ORAC value was 1567.75 μmol Trolox Equivalent/g) [33]. Additionally, the hydroxyl radical scavenging activity and GSH of walnut hydrolysate obtained by pepsin were 31.01% and 34.84%, respectively [33]. Furthermore, the ORAC value of the hydrolysate of defatted peanut meal was 1160 μmol Trolox Equivalent/g and the value of GSH was 1350 μmol Trolox Equivalent/g [47]. 

### 4.2. In Vitro Models

The cellular models are valuable tools for assessing the antioxidant capacity of nut-derived peptides. They are a better choice than animal models and human clinical trials, considering their rapidity of execution and low cost. Moreover, cellular models give more biologically relevant information compared with chemical assays such as cellular uptake, absorption, distribution, and metabolism of antioxidant compounds [3]. Cell culture models are different, based on the type of disease to be explored and evaluated [5]. Examples of cell lines used in neuroscience studies include neuroblastoma cells (SH-SY5Y), rat pheochromocytoma cells (PC12), Neuro-2a cells, BV-2 cells, the Mus musculus hippocampal neuronal cells (HT-22), Caco-2 cells and HepG2 cells. Human breast cancer cells (MDA-MB231), human umbilical vein endothelial cells (HUVECs), and Michigan cancer foundation-7 cells (MCF-7) are often used as cell models for studying various cancers. Compared with the traditional determination of chemical antioxidant activity in vitro, the determination of cellular antioxidant activity (CAA) developed by Wolfe and Liu is a biological representative method with cellular biochemical processes [76]. The CAA value is usually expressed in micromoles of quercetin equivalent (QE) per 100 g of peptide [6]. Yang et al. reported that the CAA values of peptide Lys-Trp-Phe-Cys-Thr and Gln-Trp-Phe-Cys-Thr were 612.8 and 916.3 µmol QE/100 g, respectively, which were identified from pine nut (*Pinus koraiensis*) powder protein hydrolyzed by alkaline protease [70]. H_2_O_2_ can pass through biological membranes freely and can be easily transformed into highly active hydroxyl radicals in cells [77]. Therefore, H_2_O_2_ is often used to induce oxidative stress in cellular models in order to study the antioxidant properties of nut-derived antioxidant peptides. It was reported that defatted walnut meal protein hydrolysates at 0.1 mg/mL displayed protective cell ability against H_2_O_2_-induced PC12 cell [26], and that cell viability was enhanced at the concentration of 50 μg/mL of the peanut peptide Tyr-Gly-Ser in H_2_O_2_-induced PC12 cell [47]. Moreover, either the defatted walnut meal protein hydrolysates or the peanut peptide Tyr-Gly-Ser were more efficient than GSH in reducing the H_2_O_2_-induced cellular damage [26,47]. These results showed that cell viability induced by H_2_O_2_ can be used as a preliminary index to evaluate the antioxidant activity of peptides from nut proteins.

In addition, intracellular enzymatic and non-enzymatic defense systems are key in scavenging superoxide and hydroxyl radicals in biological systems. For this reason, they have become important parameters to monitor in cellular models [78]. The cellular assays used for assessing the antioxidant properties of peptides derived from nut proteins are listed in Table 4. Ren et al. suggested that walnut hydrolysate peptide (<3 kDa fraction) decreased the level of ROS and apoptosis, and increased the content of glutathione peroxidase (GSH-Px) in H_2_O_2_-induced PC12 cells [26]. Moreover, the content of related enzymes, such as superoxide dismutase (SOD), catalase (CAT), GSH-Px, and malondialdehyde (MDA), was reversed with peptide Gln-Asp-His-Cys-His from pine nut (*Pinus koraiensis*) in H_2_O_2_-induced HepG2 cells [67].Moreover, hazelnut (*Corylus heterophylla* Fisch) hydrolase peptides reversed the level of enzymes such as CAT, GSH-Px, SOD, and heme oxygenase-1 (HO-1), and increased the content of ROS in Ang II-induced HUVECs cells [39,79].

### 4.3. In Vivo Models

For each nutrient or bioactive compound, specific in vitro models must be tailored, changed, tested, and validated against in vivo studies before any conclusion is drawn from their results [79]. It cannot, therefore, be taken for granted that an in vitro assay will yield results applicable to the in vivo situation. Therefore, whenever possible, in vivo studies from nut-derived antioxidant peptides should be used for the validation of in vitro models. The zebrafish, mice (Kunming, C57BL/6, and BALB/c), Sprague Dawley (SD) rat, and transgenic mice are commonly used as the animal models. In the animal model, biomarkers of lipid and protein peroxidation, and enzymatic and non-enzymatic systems are used to monitor change in oxidative stress in vivo. Table 5 summarizes the antioxidant effects of peptides from the nut proteins in vivo. Walnut protein hydrolysate (666 mg/kg body weight) could improve learning and memory in lipopolysaccharide-induced mice, by reversing the content of SOD, MDA, and CAT [27]. Furthermore, Zhao et al. showed that walnut-derived peptide Tyr-Val-Leu-Leu-Pro-Ser-Pro-Lys (60 mg/kg body weight) with high antioxidant activity could reverse neurotoxicity through decreased levels of LPO, 8-OHdG, and protein carbonyl, oxidative stress markers in the hippocampus of scopolamine-induced mice [82]. This may be because the antioxidant peptides in nuts can reduce the level of ROS caused by oxidative stress, improve the activity of antioxidant enzymes in the body, and then resist the extensive oxidation of biological macromolecules, thereby restoring the structure and function of cells. In brief, these data indicated that the nut-derived peptides may inhibit the impairment of the antioxidant defense system in vivo. However, to date, there are only a few studies that have evaluated the efficacy of antioxidant peptides in nut proteins, using animal models. Thus, the bioactive activity of antioxidant peptides from nut proteins should be further investigated for confirmation in animal experiments and clinical trials.

## 5. Purification and Identification of Antioxidant Peptides 

**Figure 2:**
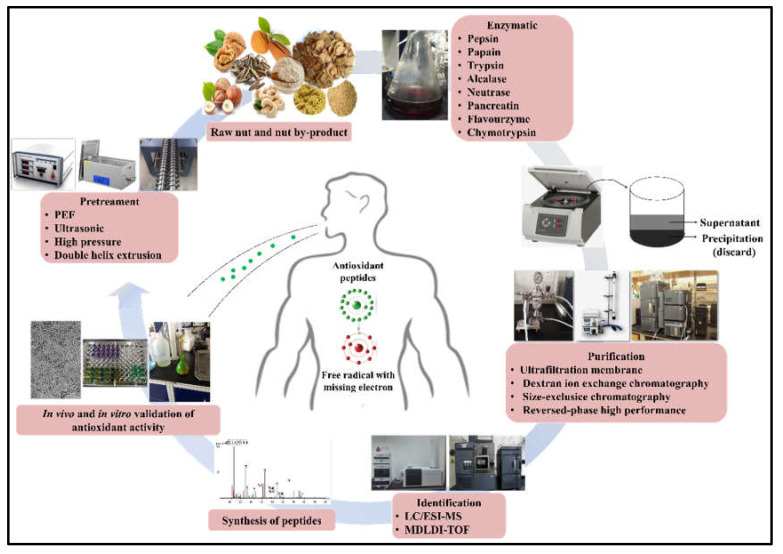
The schematic diagram for the production of antioxidant peptides from nut proteins. Abbreviations: PEF, pulsed electric field; LC-MS/MS, liquid chromatography tandem mass spectrometry; MALDI-TOF, matrix-assisted laser desorption/ionization time of fight.

Together with antioxidant peptides, protein hydrolysates also contain pro-oxidant peptides, which may decrease or nullify the total antioxidant capacity of the protein hydrolysates [38]. Therefore, as shown in Figure 2, antioxidant peptides should be preserved, whereas pro-oxidant peptides should be discarded, to enhance their total antioxidant capacity. Additionally, it is necessary to isolate a single bioactive peptide to understand the structural characteristics of peptide and further improve the antioxidant activity of final products for application and commercialization. 

The experimental techniques commonly used by researchers to purify and identify antioxidant peptides from nut protein hydrolysates are similar to those often used to find other bioactive peptides. The purification of antioxidant peptides from nut protein hydrolysates is closely related to the physical and chemical properties of the peptides, and aided by chromatographic and non-chromatographic peptide separation techniques. Peptides with different MW can be separated by ultrafiltration membrane systems with different cut-off sizes (10, 5, 3, and 1 kDa) or size-exclusion chromatography (SEC) (such as Sephadex G-75, G-25, G-15, and G10). Ion-exchange chromatography separates peptides according to their positive and negative charges. The hydrophilic and hydrophobic peptides are isolated by reversed-phase high-performance liquid chromatography (RP-HPLC). To obtain a highly purified fraction or peptide, combined purification methods should be used in sequence. Meanwhile, chemical methods and cellular models are used to detect the antioxidant activity of the purified fraction. Eventually, the amino acid sequence of the peptide is identified by the fraction with the strongest antioxidant capacity, through either liquid chromatography-tandem mass spectrometry detection (LC-MS/MS) or matrix-assisted laser desorption/ionization-time of flight (MALDI-TOF) and electron spray ionization (ESI) mass spectrometry analysis. Finally, once the peptide sequence is known, the peptide can be synthesized by a chemical method to verify its antioxidant activity and carry out additional characterization. 

Liu et al. purified walnut hydrolysate by SEC (Sephadex G-25 and Sephadex G-15), RP-HPLC, and HPLC-MS/MS, and demonstrated that one of the identified peptides, Glu-Val-Ser-Gly-Pro-Gly-Leu-Ser-Pro-Asn, reduced the level of ROS, elevated cell viability and the activities of CAT, SOD and GSH-Px in H_2_O_2_-induced PC12 cells [38]. Lin et al. obtained the pine nut (Pinus koraiensis)-derived antioxidant peptide fractions (3–10 kDa) through Sephadex gel filtration chromatography and HPLC-MS/MS [69]. In addition, due to the rapid development of biotechnology, the existence of various post-translational modifications found on certain amino acids could bring additional challenges to peptide identification [6]. At present, most of the nut-derived antioxidant peptides identified and reported in the literature have unmodified amino acid residues. The possibility of identification of nut-derived antioxidant peptides composed of modified residues certainly attracts attention for future research.

## 6. Structure-Activity Relationship of Antioxidant Peptides

SAR is a recognized tool for chemical activity evaluation, which has been widely used in the prediction of bioactive peptides [89], such as antioxidant activity [90], or ACE-inhibitory peptides [91]. Obtaining the SAR of antioxidant peptides is of great significance for providing guidance and predicting the evaluation based on amino acid sequence and enzyme cleavage bond. However, only a few studies have evaluated the SAR of antioxidant peptides in nut proteins. The exact mechanism of the antioxidant activity of peptides is not fully understood, but several common characteristics of peptides with considerable antioxidant capacity have emerged. In general, the antioxidant activity of peptides is determined by the amino acid compositions, sequences, MW, and structures [92], as will be described below.

### 6.1. Molecular Weight

**Figure 3:**
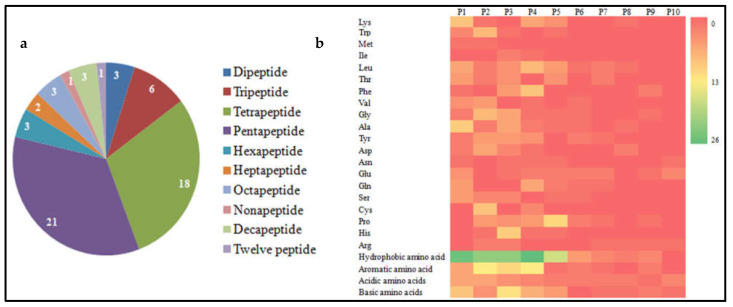
Characterization of antioxidant peptides from nut proteins. (**a**) The pie chart shows the proportions of the identified antioxidant peptides. The numbers on the pie chart indicate the number of each type of peptide. (**b**) Heat map of the frequency of amino acid residues occurring at the residues located from the first position (P1) to the tenth position (P10) of the N-terminus amino acid of the identified antioxidant peptides.

Several researchers have tried to demonstrate and explain the relationship between MW and antioxidant activity. Figure 3a and Table 6 show 61 antioxidant peptides extracted from nut proteins reported in the literature. These nut-derived peptides with antioxidant activity contain 2–12 residues with an MW ranging from approximately 282 Da and 1520 Da, with 2–5 amino acids accounting for 78.69%. This is generally consistent with other research results, that is, the MW of antioxidant peptides extracted from plant and non-plant foods ranges from 250 to 1800 Da [6]. Smaller MW peptides are generally considered to have stronger antioxidant activity than larger peptides. For example, Chinese chestnut (*Castanea mollissima* Blume)-derived peptide Val-Tyr-Thr-Glu (590.2 Da) has higher ABTS radical scavenging capacities than peptide Met-Met-Leu-Gln-Lys (745.29 Da) [54]. Small MW peptides may more easily enter the peripheral blood through the gastrointestinal (GI) barrier and blood brain barrier, and promote their bioactive activities at the tissue level.

### 6.2. Constitution and Sequence of Amino Acid

The effect of amino acid composition on the antioxidant activity of peptides is well described in the literature, indicating the fact that amino acids exert unique antioxidant activity, according to the properties of their side residues. The analysis of the amino acid compositions of nut-derived antioxidant peptides revealed that the percentage of the peptide containing hydrophobic amino acids reached 88.52% (Table 6), as the hydrophobicity of peptides can promote the existence of peptides at the water-lipid interface, and favors the scavenging of radicals from the lipid phase [30,89]. Meanwhile, hydrophobic peptides can easily cross the cell membrane in living cells, promote the solubility of peptides in lipids, and enhance the contact with hydrophobic free radicals [93]. Wang et al. reported a peptide Leu-Leu-Pro-Phe, which contains four consecutive hydrophobic amino acids, and found that it possesses extremely high ABTS scavenging activity [28]. As shown in Table 6, the number of nut-derived antioxidant peptides containing aromatic amino acids was 39 (accounting for more than 50%), and since aromatic amino acids (including Phe, Trp, Tyr, and His) provide protons for electron-deficient radicals, they contribute to the radical scavenging properties of amino acid residues [49]. These results indicate the importance of aromatic amino acids, as well as hydrophobic amino acids, in antioxidant peptides. Moreover, there were 29 peptides which contained basic amino acid residues and 19 peptides which contained acidic amino acids (Table 6). The acidic amino acids (Asp and Glu) and basic amino acids (His, Arg, and Lys) can quench unpaired electrons and radicals by using carbonyl and amino groups in the side chain as chelators of metal ions [94]. Liu et al. reported that hazelnut-derived antioxidant peptide with an amino sequence of Asp-Trp-Asp-Pro-Lys involving two acidic amino acids, one basic amino acid, one aromatic amino acid, and two hydrophobic amino acids, possessed high ABTS and DPPH scavenging capacity [39]. 

Figure 3b shows the frequency of amino acid residues located from the first position (P1) to the tenth position (P10) at the N-terminus of the identified nut-derived antioxidant peptides. The residues at P1-P9 (especially P1-P5) of these peptides are mainly hydrophobic amino acids (such as Val-Leu-Leu-Ala-Leu-Val-Leu-Leu-Arg), in which basic amino acids and acidic amino acids account for 52.46% and aromatic amino acids (> 50%). Therefore, the activity of the antioxidant peptide is not only correlated with the length of the peptide chain, but its amino acid composition appears to make a greater contribution to the antioxidant effect of the peptide. The hydrophobic amino acids, especially when present at the C- or N-terminal, such as Val in Val-Tyr-Tyr [28] and Val-Tyr-Thr-Glu, and Val-Ser-Ala-Phe-Leu-Ala [54], Trp in Trp-Ser-Arg-Glu-Glu-Gln-Glu-Arg-Glu-Glu [29], and Gly-Gly-Trp [28] and Ala-Pro-Thr-Leu-Trp [27], as well as basic and acidic amino acids in the peptides, make a significant contribution to antioxidant properties. Basic amino acids, especially His at P3, were found to possess high antioxidant capacity, which may be due to the ability of donating hydrogen ions, capturing lipid peroxyl radicals, and chelating metal ions of imidazole ring in the R group [3,30].

### 6.3. Structure of Antioxidant Peptide 

Some studies have explored the relationship between the spatial structure and secondary conformation of peptides derived from nut proteins and their antioxidant activities. All identified peptides possessed more than one folding pattern with a well structure, and the cellular antioxidant capacity was negatively correlated with the content of α-helix and random coil. Liang et al. reported that pulsed electric field (PEF) was used to treat the pine nut-derived peptide Gln-Asp-His-Cys-His, and found that, compared with the untreated peptide, the PEF-treated peptide increased the level of CAT, SOD, GPx, and GR, and reduced the level of MDA in H_2_O_2_-induced HepG2 cells [67]. The CD test showed that the decline of the α-helix and random coil contents of Gln-Asp-His-Cys-His after treated by PEF [69]. Similarly, Yang et al. reported that DPPH and ABTS free radical scavenging abilities of pine nut (*Pinus koraiensis*) peptide were improved with PEF treatment, and it was also observed that the changing trend of antioxidant activity was opposite to that of random coil content [70]. Additionally, PEF treatment also reduced zeta potential and size distribution, changed the spatial configuration of peptide Lys-Asp-His-Cys-His, and enhanced DPPH, ABTS and hydroxyl radical scavenging capacity through the change in left chemical shift of active hydrogen in hydroxyl [95]. These findings suggest that PEF treatment of the peptide solution led to a structural change, which may have enhanced the exposure of active sites responsible for the antioxidant activity of the peptide.

## 7. Bioavailability of Nut-Derived Antioxidant Peptides

The beneficial effects that antioxidant peptides may exert on the human body depend on stability and integrity of the peptides through uptake, metabolism, and biological distribution, even before considering dose- and host-related factors; that is, these antioxidant peptides must be bioavailable before exerting their bioactivity. Therefore, it is necessary to verify whether antioxidant peptides can resist the degradation of GI enzymes after entering the digestive system. Data from in vivo assays constitute the reference standard and provide the highest scientific evidence for the bioavailability of antioxidant peptides [96]. However, the limitations in experimental design, difficulties in data interpretation, high equipment and labor costs, and ethical constraints, limit the application of in vivo methods for screening the bioavailability of antioxidant peptides [97]. On the contrary, in vitro models are rapid, cost-effective and reproducible methods that can be used to determine the bioavailability of antioxidant peptides. Among them, the Caco-2 cell model is the validated intestinal epithelial cell model, which is widely used to detect digestion in vitro.Despite little information being available on the bioavailability of antioxidant peptides from nut proteins, there is a report that Chinese chestnut-derived antioxidant peptides Val-Tyr-Thr-Glu, Thr-Lys-Gly-Gln, Met-Met-Leu-Gln-Lys, and Thr-Pro-Ala-Ile-Ser show only a slight change in ABTS radical scavenging capacity upon digestion with a mix of porcine pepsin, trypsin, and pancreatin, indicating that these peptides retain well their antioxidant activity in the GI system [54]. Similar results were reported on the stability of antioxidant peptide Gln-Asp-His-Cys-His in simulated GI digestion from pine nut (*Pinus koraiensis*) [98]. These results indicate that these nut-derived antioxidant peptides retain well their activity in the GI system. Nevertheless, although in vitro simulation of nut-derived antioxidant peptides has potential and wide applicability, it does not fully mimic the overall processes occurring in vivo, especially hormonal and nervous control, feedback mechanisms, mucosal cell activity, the complexity of peristaltic movements, and involvement of the local immune system. In addition, there is an urgent need to standardize in vitro methods, to afford improved research designs closer to the situation in vivo.

## 8. Conclusions

The search for natural antioxidants to replace synthetic equivalents has always been an important activity among the international research community. Although past studies have demonstrated that nut-derived antioxidant peptides have high nutritional value because of their antioxidant properties and are expected to be potential substitutes for synthetic antioxidants or used in the development of human nutritional functional foods, few marketable products are available so far. This may be due to various reasons, including a lack of in vivo and clinical trials (to confirm biological activity, bioavailability, efficacy and safety), high production costs, difficulties in the standardization of the production process, and the presence of potential nut-derived allergens. In addition, the MW, composition, and sequence of amino acids have the greatest impact on the antioxidant activity, as presented here in Section 6. The bioinformatics method of SAR has shown significant advantages in evaluating nut-derived antioxidant peptides, but its development is still in its infancy. With the help of a deeper SAR knowledge, the work of exploring antioxidant peptides will become a systematic, simple, and designed procedure. 

## Figures and Tables

**Table 1:** Protein fractions, limiting amino acid and digestibility of nuts.

	Albumins (%)	Globulins (%)	Prolamins (%)	Glutenins (%)	Limiting Amino Acids	Crude Protein Content (%)	Defatted Protein Content (%)	Digestibility (%)
Walnut	6.80	17.60	5.30	70.10	Met + Cys, Lys	18.00	55.96	86.22
Pine nut	52.54	15.16	7.64	4.95	Met + Cys, Val	31.10	47.69	88.50
Hazelnut	67.18	17.62	3.17	6.53	Lys, Leu	15.30	58.80	-
Almond	75.43	13.63	5.75	5.18	Met + Cys, Lys	23.70	40.74	73.52
Brazil nut	17.70	6.00	-	3.00	-	14.50	46.00	70.31
Cashew nut	7.69	17.30	-	7.80	Lys	20.20	40.74	69.71
Pistachio	25.00	66.00	2.00	7.30	-	29.60	41.04	-
Macadamia	2.39	11.62	-	4.45	Met	7.76	25.50	75.70
Pecan	2.04	22.13	9.00	64.94	Met + Cys, Lys	10.00	34.80	-
Peanut	-	-	-	-	Met, Leu	22.30	47.90	70.00

**Table 2:** Antioxidative hydrolysate of nut protein prepared by enzymatic methods.

Protein Sources	Bioactivities	Enzymes	Hydrolysis Conditions	Reference
Defatted walnut meal	Antioxidant	Pancreatin, Pepsin	Pepsin/substrate ratio of 1:10 (*w*/*w*), pH 2.0, 37 °C, 3 h; Pancreatin/substrate ratio of 1:10 (*w*/*w*), 37 °C, pH 7.4, for another 3 h.	[12]
Defatted peanut flour	Antioxidant, ACE-inhibitory	Alcalase	Alcalase/substrate ratio of 1:10 (*w*/*w*), 50 °C, 22 h, pH 8.0.	[22]
Walnut (*Juglansmand shurica* Maxim.) protein isolate	Antioxidant, improve learning and memory	Neutrase, Alcalase	Neutrase: 9000 U/g, pH 7.0, 52.5 °C; Alcalase: 7000 U/g, pH 8.4, 55.5 °C.	[26]
Walnut (*Juglans regia*)	Antioxidant, neuroinflammation	Pancreatin, Viscozyme L	Viscozyme L (protease/substrate 1.0%, *w*/*w*) and pancreatin (protease/substrate 1.0%, *w*/*w*), pH 7.0, 55 °C, 12 h.	[27]
Walnut (*Juglans regia*) protein power	Antioxidant, reverse sleep deprivation	Pancreatin, complex plant hydrolase	Enzyme/substrate ratio of 1.0%, pH 7.0, 55 °C, 12 h.	[28]
Defatted walnut meal	Antioxidant, improve learning and memory	Pancreatin	Pancreatin/substrate ratio of 20:1 (*w*/*w*), pH 8.0, 55 °C, 12 h.	[29]
Walnut (*Juglans regia* L.) protein	Antioxidant	Neutrase, Papain, Bromelain, Alcalase, Pepsin, Pancreatin	Neutrase: 50 °C, pH 7.0, 4 h, ratio 1:30; Papain: 50 °C, pH 7.0, 4 h, ratio 2:30; Bromelain: 50 °C, pH 7.0, 4 h, ratio 3:30; Alcalase: 50 °C, pH 8.0, 4 h, ratio 3:30; Pepsin: 37 °C, pH 2.0, 4 h, ratio 1:30; Pancreatin: 50 °C, pH 8.0, 4 h, ratio 2:30.	[30]
Walnut (*Juglans regia* L.) protein	Antioxidant, anticancer	Chymotrypsin, Trypsin, Proteinase K	Enzymes/substrate ratio of 1:100 (*w*/*w*).	[31]
Walnut (*Juglans Sigillata* Dode) meal proteins	Antioxidant	Pancreatin	Pancreatin/substrate ratio of 2:100 (*w*/*w*), pH 7.5, 37 °C, 22 h.	[32]
Walnut (*Juglans regia* L.) protein	Antioxidant	Neutrase, Alcalase, Pepsin	Neutrase: pH 7.0, 50 °C, 0.5 h; Alcalase: pH 8.0, 50 °C, 0.5 h; Pepsin: pH 2.0, 37 °C, 3 h.	[33]
Defatted walnut meal	Antioxidant, improve learning and memory	Pancreatin, Viscozyme L	Enzyme mixture of pancreatin and viscozyme L/substrate ratio of 8:1000 (*w*/*w*), 55 °C, 16 h.	[34]
Walnut (*Juglans regia*)	Antioxidant, hyperuricemia	Alcalase	Alcalase/substrate ratio of 1:100 (*w*/*w*), pH 7.0, 99 °C, 9 h.	[35]
Walnut meal	Antioxidant, antihypertensive	Alcalase, Trypsin	Alcalase/substrate ratio of 6:100 (*w*/*w*), pH 9.5, 60 °C, 2.5 h; Trypsin/substrate ratio of 6:100, pH 8.0, 37 °C, 3.5 h.	[36]
Walnut meals	Antioxidant, antiproliferative	Trypsin	Trypsin/substrate ratio of 1:100 (*w*/*w*), 55 °C, 22 h.	[37]
Defatted walnut meal	Antioxidant, ACE inhibition	Alcalase	Alcalase/substrate ratio of 2:100 (*w*/*w*), 50 °C, pH 9.0.	[38]
Hazelnut (*Corylus heterophylla* Fisch) protein	Antioxidant	Alcalase	10,000 U/g, 54 °C, pH 8.0, 2.5 h.	[39]
Pine nut (*Pinus gerardiana*)	Antioxidant, ACE-inhibitory	Alcalase	Alcalase/substrate ratio of 7:100 (*w*/*w*), pH 8.5, 55 °C, 3 h.	[40]
Peanut kernel (*Arachis hypogaea* L.)	Antioxidant	Esperase, Neutrase	Esperase/substrate ratio of 1:200 (*w*/*w*), pH 8.0, 60 °C, 2 h. Neutrase/substrate ratio of 1:200 (*w*/*w*), pH 6.0, 50 °C, 24 h.	[41]
Defatted peanut meal	Antioxidant	Protease	Protease/substrate ratio of 4:1000 (*w*/*w*), pH 6.7, 55 °C, 24 h.	[42]
Peanut meal	Antioxidant	Alcalase, Flavourzyme, L-cysteinase, Neutral	Peanut meal (5% *w*/*w*) were preheated to optimal temperatures and pH of papain (60 °C, pH 7.0), flavourzyme (60 °C, pH 7.5), L-cysteinase (55 °C, pH 4.5), Alcalase and neutral complex protease (55 °C, pH 8.5), respectively. The temperature was maintained for 3 h to hydrolyze.	[43]
Defatted peanut	Antioxidant	Alcalase	Alcalase/substrate ratio of 5:100 (*w*/*w*), pH 8.0, 53 °C, 8 h.	[44]
Defatted peanut flour	Antioxidant	Alcalase	Alcalase/substrate ratio of 5:100 (*w*/*w*), pH 8.0, 50 °C, 1 h.	[45]
Defatted peanut protein powder	Antioxidant	Papain	The papain (5000 u/g) 50 °C, 15 min, pH 8.0.	[46]
Defatted peanut meal	Antioxidant	Protease	Protease/substrate ratio of 4:1000 (*w*/*w*), 55 °C, 12 h, pH 6.7.	[47]
Defatted peanut cake powder	Antioxidant	Alcalase	Substrate mass fraction was 10%, enzyme dosage of 4000 U/g, pH 8.5, 60 °C, 25 min.	[48]
Defatted peanut flour	Antioxidant	Alcalase	Alcalase/substrate ratio of 1:250 (*w*/*w*), 60 °C, 3 h, pH 8.0.	[49]
Defatted peanut cake	Antioxidant	Alcalase, Pepsin	Alcalase/substrate ratio of 1:25 (*w*/*w*), 60 °C, pH 8.0.Pepsin/substrate ratio of 8:5 (*w*/*w*), 37 °C, pH 2.0.	[50]
Bambara groundnut (*Vigna subterranea*)	Antioxidant	Alcalase, Trypsin, Pepsin	Enzymes/substrate ratio of 1:100 (*w*/*w*), 4 h for Alcalase 50 °C, pH 8.0; Trypsin 37 °C, pH 8.0; Pepsin 37 °C, pH 2.0.	[51]
Bambara groundnut seeds	Antioxidant, ACE-inhibitory	Alcalase, Trypsin, Pepsin	Enzymes/substrate ratio of 1:100 (*w*/*w*), 4 h for Alcalase 50 °C, pH 8.0; Trypsin 37 °C, pH 8.0; Pepsin 37 °C, pH 2.0.	[52]
Wild almond (*Amygdalus scoparia*)	Antioxidant	Pepsin, Chymotrypsin, Trypsin, Alcalase, Flavourzyme	Enzymes/substrate ratio of 1:100 (*w*/*w*), Pepsin, chymotrypsin and trypsin (37 °C, pH 7.8), Alcalase (50 °C, pH 8.0), flavourzyme (50 °C, pH 7.0) for 3 h.	[53]
Chinese chestnut (*Castanea mollissima* Blume)	Antioxidant	Alcalase	Alcalase/substrate ratio of 3:100 (*w*/*w*), 55 °C, pH 10.0, 4 h.	[54]
Defatted pecan seed meals	Antioxidant	Alcalase	Alcalase/substrate ratio of 1:20 (*w*/*w*), 55 °C, pH 10.0, 3 h.	[55]
Sunflower	Antioxidant, antimicrobial	Alcalase, Flavourzyme	Alcalase/substrate ratio of 1:10 (*w*/*w*), 58 °C, pH 8.0, 3 h. Flavourzyme/substrate ratio of 3:500 (*w*/*v*), 50 °C, pH 6.5, 2 h.	[56]

**Table 3:** Evaluation of antioxidant activity of nut-derived peptides by chemical methods.

Protein Sources	Bioactivities	Chemical Assay	Reference
Defatted walnut meal	Antioxidant	ABTS, ORAC	[12]
Defatted peanut flour	Antioxidant, ACE-inhibitory	Reducing power, DPPH, MCA, β-carotene bleaching assay	[22]
Walnut (*Juglansmand shurica* Maxim.) protein isolate	Antioxidant, improve learning and memory	ORAC, hydroxyl radical scavenging activity, FRAP	[26]
Defatted walnut meal	Antioxidant, improve learning and memory	Hydroxyl radical scavenging activity, ORAC	[29]
Walnut (*Juglans regia* L.) protein	Antioxidant	DPPH, ABTS, SRSA	[30]
Walnut (*Juglans regia* L.) protein	Antioxidant, anticancer	ABTS	[31]
Walnut (*Juglans Sigillata* Dode) meal proteins	Antioxidant	DPPH, ABTS, FRAP, ORAC	[32]
Walnut (*Juglans regia* L.) protein	Antioxidant	DPPH, hydroxyl radical scavenging, FRAP, reducing power, inhibition of linoleic acid peroxidation	[33]
Defatted walnut meal	Antioxidant, improve learning and memory	Reducing power, ORAC, hydroxyl radical, radical-scavenging activity, ABTS	[34]
Walnut meal	Antioxidant, antihypertensive	Reducing power, DPPH, lipidperoxidation	[36]
Defatted walnut meal	Antioxidant, ACE inhibition	DPPH	[38]
Pine nut (*Pinus gerardiana*)	Antioxidant, ACE-inhibitory	DPPH, reducing power	[40]
Peanut kernel (*Arachis hypogaea* L.)	Antioxidant	DPPH, MCA, reducing power	[41]
Defatted peanut meal	Antioxidant	ORAC	[42]
Peanut meal	Antioxidant	Reducing power, DPPH	[43]
Defatted peanut	Antioxidant	Reducing power, DPPH	[44]
Defatted peanut flour	Antioxidant	Reducing power, DPPH, hydroxyl radical-scavenging activity	[45]
Defatted peanut protein powder	Antioxidant	Reducing power, DPPH, hydroxyl radical-scavenging activity, antioxidative activity	[46]
Defatted peanut meal	Antioxidant	ORAC, DPPH, reducing power, MCA, lipid peroxidation	[47]
Defatted peanut cake powder	Antioxidant	SRSA, DPPH, hydroxyl radical scavenging activity, iron reduction capacity, FRAP, molybdenum reduction capacity, copper ion chelation, anti-lipid peroxidation activity	[48]
Defatted peanut flour	Antioxidant	Reducing power	[49]
Defatted peanut cake	Antioxidant	DPPH	[50]
Bambara groundnut (*Vigna subterranea*)	Antioxidant	DPPH, SRSA, hydroxyl radical scavenging assay, MCA, ferric reducing power assay	[51]
Bambara groundnut seeds	Antioxidant, ACE-inhibitory	Inhibition of linoleic acid oxidation, ABTS	[52]
Wild almond (*Amygdalus scoparia*)	Antioxidant	Reducing power, ABTS	[53]
Chinese chestnut (*Castanea mollissima* Blume)	Antioxidant	ABTS, DPPH, hydroxyl radical scavenging	[54]
Defatted pecan seed meals	Antioxidant	ABTS, DPPH, hydroxyl radical scavenging, FRAP, reducing power	[55]
Sunflower	Antioxidant, antimicrobial	Reducing power, DPPH	[56]
Walnut protein	Antioxidant	ORAC	[60]
Defatted walnut (*Juglans regia* L.) Meal	Antioxidant	Hydroxyl radical scavenging	[61]
Walnut protein meal	Antioxidant	DPPH, free radical scavenging, FRAP	[62]
Walnut meal	Antioxidant, ACE-inhibitory	Hydroxyl radicals scavenging, SRSA, DPPH, reducing power	[63]
Hazelnut (*Corylus avellana* L.) meal	Antioxidant, antiproliferative, antihypertensive	ORAC, FRAP	[64]
Pine nut (*Pinus koraiensis*) protein	Antioxidant	DPPH, ORAC	[65]
Pine nut (*Pinus koraiensis)*	Antioxidant	DPPH	[66]
Pine nut (*Pinus koraiensis)*	Antioxidant	DPPH, ABTS	[67]
Pine nut (*Pinus koraiensis)*	Antioxidant	DPPH, ABTS	[68]
Pine nut (*Pinus koraiensis)*	Antioxidant	DPPH	[69]
Pine nut (*Pinus koraiensis*) meal protein	Antioxidant	DPPH, ABTS, FRAP	[70]
Pine nut (*Pinus koraiensis*)	Antioxidant	Hydroxyl radical scavenging capacity, FRAP	[71]
Peanut meal	Antioxidant	SRSA, DPPH, MCA, reducing power, inhibition of linoleic acid autoxidation	[72]
Peanut meal	Antioxidant	Reducing power, DPPH, hydroxyl radical-scavenging activity, MCA	[73]
Peanut meal	Antioxidant	SRSA, DPPH, ABTS, inhibition of linoleic acid autoxidation, reducing power, MCA	[74]
Apricot seed kernels	Antioxidant	Hydroxyl radical scavenging, DPPH	[75]

Abbreviations: ORAC, oxygen radical absorbance capacity; FRAP, ferric reducing antioxidant power; DPPH, 2,2-diphenyl-1-picrylhydrazyl; ABTS, 2,2′-azino-bis-(3-ethylbenzothiazoline-6-sulphonic acid); SRSA, superoxide radical scavenging assay; MCA, metal chelating activity.

**Table 4:** Evaluation of antioxidant activity of nut-derived peptides by cellular models.

Protein Sources	Bioactivities	Cellular Model	Reference
Walnut (*Juglansmand shurica* Maxim.) protein isolate	Antioxidant, improve learning and memory	H_2_O_2_-induced PC12 cells: ROS, GSH-Px	[26]
Walnut (*Juglans regia*)	Antioxidant, neuroinflammation	LPS-elicited inflammation in BV-2 cells: ROS	[27]
Walnut (*Juglans regia*) protein power	Antioxidant, reverse sleep deprivation	Glutamate-induced PC12 cells: ROS, GSH-Px, SOD, MDA	[28]
Defatted walnut meal	Antioxidant, improve learning and memory	H_2_O_2_-injured PC12 cells: ROS	[29]
Walnut (*Juglans regia* L.) protein	Antioxidant, anticancer	HT-29 and MDA-MB231 tumor cells	[31]
Walnut (*Juglans Sigillata* Dode) meal proteins	Antioxidant	H_2_O_2_-induced PC12 cells	[32]
Walnut meals	Antioxidant, antiproliferative	MCF-7 cells: ROS	[37]
Hazelnut (*Corylus heterophylla* Fisch) protein	Antioxidant	Ang II-treated HUVECs cells: ROS, SOD, XO-1, HO-1	[39]
Defatted peanut meal	Antioxidant	H_2_O_2_-induced PC12 cells	[47]
Walnut protein	Antioxidant	H_2_O_2_-injured SH-SY5Y cells: ROS	[60]
Defatted walnut (*Juglans regia* L.) meal	Antioxidant	H_2_O_2_-induced SH-SY5Y cell	[61]
Pine nut (*Pinus koraiensis)*	Antioxidant	HepG2 cells: CAA	[66]
Pine nut (*Pinus koraiensis)*	Antioxidant	H_2_O_2_-induced HepG2 cells: CAA, T-SOD, CAT, GSH-Px, GSH-Rx, MDA	[67]
Pine nut (*Pinus koraiensis*) meal protein	Antioxidant	HepG2 cells: CAA	[70]
Walnut (*Juglansmand shurica* Maxim.) protein isolate	Antioxidant, improve neurotoxicity	H_2_O_2_-induced PC12 cells: ROS, GSH-Px, SOD, CAT	[80]
Walnut (*Juglansmand shurica* Maxim.) protein isolate	Antioxidant, neuroprotection	Aβ_25–35_-induced PC12 cells: ROS, GSH-Px, ATP, apoptosis	[81]
Walnut (*Juglansmand shurica* Maxim.) protein isolate	Antioxidant, improve learning and memory	H_2_O_2_-induced HT-22 cells: ROS, apoptosis, ATP, Mito SOX	[82]
Walnut (*Juglansmand shurica* Maxim.) protein	Antioxidant, antidiabetic	Glucose-induced HepG2 cells: ROS, GSH-Px, SOD, CAT	[83]
Walnut (*Juglans regia* L.) protein	Antioxidant, neuroprotective	H_2_O_2_-induced PC12 cells	[84]
Walnut (*Juglansmand shurica* Maxim.) protein isolate	Antioxidant, Anti-inflammation	LPS-injured BV-2 cells: ROS, SOD, CAT	[85]
Hazelnut (*Corylus heterophylla* Fisch) protein	Antioxidant	Ang II-induced HUVECs cells: ROS, CAT, T-SOD, GSH-Px, MDA	[86]
Pine nut	Antioxidant, improving memory impairment	H_2_O_2_-induced PC12 cells: SIRT3, ace-SOD2	[87]

Abbreviations: H_2_O_2_, hydrogen peroxide; ROS, reactive oxygen species; GSH-Px, glutathione peroxidase; PC12 cells, rat pheochromocytoma cells; SOD, superoxide dismutase; CAT, catalase; ATP, adenosinetriphosphate; SH-SY5Y cells, neuroblastoma cells; MCF-7 cells, michigan cancer foundation-7 cells; Ang II, Angiotensin II; LPS, lipopolysaccharide; HO-1, heme oxygenase 1; CAA, cellular antioxidant activity; MDA, malondialdehyde; SIRT3, NAD-dependent protein deacetylase sirtuin-3.

**Table 5:** Evaluation of antioxidant activity of nut-derived peptides by animal models.

Protein Sources	Bioactivities	Animal Model	Reference
Defatted walnut meal	Antioxidant	D-gal + AlCl3-induced mice: SOD, MDA, GSH-Px	[12]
Walnut (*Juglansmand shurica* Maxim.) protein isolate	Antioxidant, improve learning and memory	Scopolamine-treated mice: SOD, GSH-Px	[26]
Walnut (*Juglans regia*)	Antioxidant, neuroinflammation	LPS-treated mice: SOD, GSH-Px, CAT, MDA	[27]
Walnut (*Juglans regia*) protein power	Antioxidant, reverse sleep deprivation	SD rats with sleep deprivation: SOD, GSH, GSH-Px, CAT, MDA	[28]
Walnut (*Juglansmand shurica* Maxim.) protein isolate	Antioxidant, improve learning and memory	Scopolamine-induced C57BL/6 mice: apoptosis, 8-OHdG, LPO, ATP, protein carbonyl	[82]
Walnut (*Juglans regia* L.) protein	Antioxidant, neuroprotective	Mycophenolate mofetil-induced zebrafish: apoptosis	[84]
Pine nut	Antioxidant, improving memory impairment	Scopolamine-induced C57BL/6 mice: SIRT3, ace-SOD2	[87]
Walnut (*Juglans regia*) Protein	Antioxidant, neuroprotective	scopolamine-induced mice: SOD, MDA, CAT, GSH-Px	[88]

Abbreviations: SOD, superoxide dismutase; GSH-Px, glutathione peroxidase; 8-OHdG, 8-hydroxy-2-deoxyguanosine; LPO, lipid peroxidation; ATP, adenosinetriphosphate; GSH, glutathione; MDA, malondialdehyde; CAT, catalase; SIRT3, NAD-dependent protein deacetylase sirtuin-3; D-gal, D-galactose.

**Table 6:** The SAR of nut-derived peptides.

Protein Sources	Bioactive	Peptide	MW (Da)	Reference
Defatted walnut meal	Antioxidant	Thr-Tyr Ser-Ser-Glu Thr-Arg-Asn Asn-Pro-Ala-Asn Ser-Gly-Gly-Tyr Ala-His-Ser-Val-Gly-Pro	282.30 321.29 387.44 414.42 382.38 566.62	[12]
Walnut (*Juglans regia*)	Antioxidant, neuroinflammation	Leu-Pro-Phe Gly-Val-Tyr-Tyr Ala-Pro-Thr-Leu-Trp	376.2234 501.2346 587.3186	[27]
Walnut (*Juglans regia*) protein power	Antioxidant, reverse sleep deprivation	Gly-Gly-Trp Val-Tyr-Tyr Leu-Leu-Pro-Phe	319.1405 444.2132 489.3067	[28]
Defatted walnut meal	Antioxidant, improving learning and memory	Trp-Ser-Arg-Glu-Glu-Gln-Glu-Arg-Glu-Glu Ala-Asp-Ile-Tyr-Thr-Glu-Glu-Ala-Gly-Arg	1377.4 1124.18	[29]
Walnut (*Juglans regia* L.) protein	Antioxidant	Ala-Asp-Ala-Phe	423.23	[33]
Hazelnut (*Corylus heterophylla* Fisch) protein	Antioxidant	Ala-Asp-Gly-Phe Ala-Gly-Gly-Phe Ala-Trp-Asp-Pro-Glu Asp-Trp-Asp-Pro-Lys Glu-Thr-Thr-Leu Ser-Gly-Ala-Phe	408.16 350.16 616.25 659.29 462.23 380.17	[39]
Defatted peanut meal	Antioxidant	Tyr-Gly-Ser	325.3	[47]
Chinese chestnut (*Castanea mollissima* Blume)	Antioxidant	Val-Tyr-Thr-Glu Thr-Lys-Gly-Gln Met-Met-Leu-Gln-Lys Thr-Pro-Ala-Ile-Ser Val-Ser-Ala-Phe-Leu-Ala	590.20 592.17 745.29 647.20 606.34	[54]
Defatted pecan seed meals	Antioxidant	Leu-Ala-Tyr-Leu-Gln-Tur-Thr-Asp-Phe-Glu-Thr-Pro	1519.75	[55]
Walnut protein	Antioxidant	Trp-Pro-Pro-Lys-Asn Ala-Asp-Ile-Tyr-Thr	640.8 710.7	[60]
Pine nut (*Pinus koraiensis*) protein	Antioxidant	Gln-Trp-Phe-His	658.72	[65]
Pine nut (*Pinus koraiensis*)	Antioxidant	Glu-Asp-His-Cys-His	621.7	[67]
Pine nut (*Pinus koraiensis*)	Antioxidant	Lys- Cys-His-Lys-Pro	611.76	[68]
Pine nut (*Pinus koraiensis*)	Antioxidant	Gln-Cys-His-Lys-Pro Gln-Cys-His-Gln-Pro Lys-Cys-His-Gln-Pro Lys- Cys-His-Lys-Pro	611.72	[69]
Pine nut (*Pinus koraiensis*) meal protein	Antioxidant	Lys-Trp-Phe-Cys-Thr Gln-Trp-Phe-Cys-Thr	683.82 683.78	[70]
Pine nut (*Pinus koraiensis*)	Antioxidant	Gln-Cys-His-Lys-Pro Gln-Cys-His-Gln-Pro Lys-Cys-His-Gln-Pro Lys- Cys-His-Lys-Pro	611.72	[71]
Walnuts (*Juglans mandshurica* Maxim.) protein	Antioxidant, improve neurotoxicity	Glu-Val-Ser-Gly-Pro-Gly-Leu-Ser-Pro-Asn	955.4611	[80]
Walnuts (*Juglans mandshurica* Maxim.) protein	Antioxidant, neuroprotection	Thr-Trp-Leu-Pro-Leu-Pro-Arg Tyr-Val-Leu-Leu-Pro-Ser-Pro-Lys Lys-Val-Pro-Pro-Leu-Leu-Tyr	882.08 916.14 829.06	[81]
Walnuts (*Juglans mandshurica* Maxim.) protein	Antioxidant, improve learning and memory	Tyr-Val-Leu-Leu-Pro-Ser-Pro-Lys	916.14	[82]
Walnut (*Juglansmand shurica* Maxim.) protein	Antioxidant, antidiabetic	Leu-Val-Arg-Leu Leu-Arg-Tyr-Leu Val-Leu-Leu-Ala-Leu-Val-Leu-Leu-Arg	499.35 563.34 -	[83]
Walnut (*Juglansmand shurica* Maxim.) protein isolate	Antioxidant, Anti-inflammation	Trp-Glu-Lys-Pro-Pro-Val-Ser-His	980.07	[85]
Hazelnut (*Corylus heterophylla* Fisch)	Antioxidant	Glu-Trp Asp-Trp-Asp-Pro-Lys Ala-Asp-Gly-Phe Ser-Gly-Ala-Phe Glu-Thr-Thr-Leu Ala-Gly-Gly-Phe	333.35 659.70 408.41 380.40 462.50 350.38	[86]
Pine nut	Antioxidant, improving memory impairment	Trp-Tyr-Pro-Gly-Lys	-	[87]
Walnut (*Juglans regia*) protein	Antioxidant, neuroprotective	Phe-Tyr Ser-Gly-Phe-Asp-Ala-Glu	329.1490 625.2451	[88]

## Data Availability

Not applicable.

## Abbreviations

•OH	hydroxyl radical
•ONOO	peroxynitrite
8-OHdG	8-hydroxy-2-deoxyguanosine
ABTS	2,2’-azino-bis (3-ethylbenzothiazoline-6-sulphonic acid)
Ang II	angiotensin II
ATP	adenosinetriphosphate
AQP	aquaporin
BHA	butylated hydroxy-anisole
BHT	butylated hydroxyl-toluene
CAA	cellular antioxidant activity
CAT	catalase
CD	circular dichroism
CoQ10	coenzymeQ 10
D-gal	D-galactose
DH	degree of hydrolysis
DPPH	2,2-diphenyl-1-picrylhydrazyl
DNA	deoxyribonucleic acid
ESI	electron spray ionization
FAO	food and agriculture organization
FRAP	ferric reducing antioxidant power
GI	gastrointestinal
GO	gene ontology
GSH	glutathione
GSH-Px	glutathione peroxidase
H_2_O_2_	hydrogen peroxide
HO-1	heme oxygenase 1
HOO•	peroxyl
HT-22	Mus musculus hippocampal neuronal cells
HUVECs	human umbilical vein endothelial cells
LC-MS/MS	liquid chromatography tandem mass spectrometry
LPO	lipid peroxidation
LPS	lipopolysaccharide
MALDI-TOF	matrix-assisted laser desorption/ionization time of fight
MCA	metal chelating activity
MCF-7	michigan cancer foundation-7 cells
MDA	malondialdehyde
MW	molecular weight
NO•	nitric oxide
NOS	nitric oxide synthase
O_2_−•	superoxide anion radical
ORAC,	oxygen radical absorbance capacity
PC12	rat pheochromocytoma cells
PEF	pulsed electric field
QE	quercetin equivalent
REDOX	oxidation-reduction
RNS	reactive nitrogen species
ROS	reactive oxygen species
RP-HPLC	reversed-phase high performance liquid chromatography
SAR	structure-activity relationship
SD	sprague dawley
SEC	size-exclusion chromatography
SH-SY5Y	neuroblastoma cells
SIRT3	NAD-dependent protein deacetylase sirtuin-3
SOD	superoxide dismutase;
SRSA	superoxide radical scavenging assay
UNU	United Nations University
WHO	World Health Organization
